# GnRH Analogues in the Prevention of Ovarian Hyperstimulation Syndrome

**DOI:** 10.5812/ijem.5034

**Published:** 2013-04-01

**Authors:** Pilar Alama, Jose Bellver, Carmen Vidal, Juan Giles

**Affiliations:** 1 Department of Reproduction, IVI-Valencia, Plaza de la Policia Local, Valencia, Spain; 2 Department of Paediatrics, Obstetrics and Gynaecology, Faculty of Medicine. University of Valencia, Valencia, Spain

**Keywords:** Ovarian Stimulation, Ovarian Hyperstimulation Syndrome, Prevention, GnRH Agonist, GnRH Antagonist

## Abstract

The GnRH analogue (agonist and antagonist GnRH) changed ovarian stimulation. On the one hand, it improved chances of pregnancy to obtain more oocytes and better embryos. This leads to an ovarian hyper-response, which can be complicated by the ovarian hyperstimulation syndrome (OHSS). On the other hand, the GnRH analogue can prevent the incidence of OHSS: GnRH antagonist protocols, GnRH agonist for triggering final oocyte maturation, either together or separately, coasting, and the GnRH analogue may prove useful for avoiding OHSS in high-risk patients. We review these topics in this article.

## 1. Context

Ovarian hyperstimulation syndrome (OHSS) is an iatrogenic complication of controlled ovarian hyperstimulation (COH) and ovulation induction.

The incidence of OHSS reported in the literature varies depending on the data collection method used, the study population and the interpretation of the classifications used (1, 2). Up to 33% of IVF cycles have been reported to be associated with mild forms of OHSS, while this percentage is about 2-6% for the severe form (3, 4).

This syndrome was first described in 1943 when early forms of gonadotropins were used to stimulate or induce ovulation, and conversely to what we may think, several reports indicate an increased incidence in severe forms and in the proportion of hospitalised patients (5). The incidence of venous thromboembolism in relation to IVF has been reported to be approximately 0.1-0.2% of treatment cycles (6). According to the data corresponding to relatively few cases, the risk of venous thromboembolism associated with OHSS is reported to be between 0.8% and 2.4% (7). Pregnancies with fresh IVF cycles complicated by OHSS were at a 100-fold increased risk of venous thromboembolism in the first trimester if compared with the background population (8). In addition to this, recent published data support a statistically significant increase in pregnancy-related complications among IVF pregnancies in women with OHSS compared to IVF controls (5, 9, 10).

The literature describes two main clinical forms of OHSS; an early form relating to the ovarian response to stimulation, which happens to be a sharp effect of exogenous hCG administration. It takes place during the first 9 days after oocyte retrieval (11, 12). The late form correlates poorly with the ovarian response: it correlates better with endogenous hCG produced by implanting an embryo or with hCG administration for luteal phase support. It occurs after the initial 10-day period and its management is clinically more difficult (12, 13).

Although the pathophysiology of this syndrome remains unknown, it is assumed that the vasoactive substances secreted by ovaries under hCG stimulation may play a key role in increasing the capillary permeability observed in OHSS.

The purpose of this paper is to review the most recent evidence in GnRH analogues paper in the prevention of ovarian hyperstimulation syndrome. A literature search was conducted by searching Pubmed for articles published in the last 5 years using the terms “ovarian hyperstimulation syndrome”, “OHSS”, “GnRH agonist”, “GnRH antagonist”, “GnRH agonist triggering”, “ovarian hyperstimulation syndrome prevention” and “ovarian hyperstimulation syndrome treatment”. For this paper were included randomized controlled trial, reptrospective studies, case studies, meta-analyses, and reviews.

## 2. Phisiopathology

To date, around 25 factors have been described as being involved in the regulation of cellular permeability (14, 15). Angiogenic cytokines including vascular endothelial growth factor (VEGF), interleukin (IL)-6, IL-8, basic fibroblast growth factor (bFGF), tumor necrotic factor-α (TNF-α), IL-1β, etc produced by multiple corpora luteae may be involved in OHSS (16, 17).

Of these, (VEGF) is one of the most important factor, while the effects of the others angiogenic factors on OHSS are vague (18).

VEGF (also referred to as VEGF-A) belongs to a gene family that includes the placenta growth factor (PlGF)(19-21), VEGF-B (22), VEGF-C (23) and VEGF-D (24).

After hCG administration, VEGF up-regulates during ovarian stimulation and has a strong permeability effect on endothelial cells. For this reason, VEGF is considered a possible candidate in relation to the increased permeability observed in OHSS with loss of fluid to the third space (25).

The potential role of the VEGF/VEGF-receptor (VEGF-R1 and R2) system in the appearance of OHSS is based on certain evidence: a) it has been demonstrated that VEGF increases the vascular permeability of endothelial cells, which could lead to a shift in fluids from the blood vessels to the third space (26-28); b) vascular permeability increased after hCG during ovarian stimulation; c) the occurrence of OHSS is strongly correlated with the application of hCG during ovarian stimulation, leading to an increased VEGF expression by granulosa cells from corpora lutea (29-31) d) In OHSS patients, the amount of VEGF in the follicular fluid is frequently higher than in persons not affected by this complication (32), thus also proving the ovarian origin of VEGF; e) the VEGF-R2 expression increases at the same time that the vascular permeability peaks, demonstrating that the VEGF-VEGF receptor system is implicated in OHSS (27, 28).

More studies are needed to understand the real role of the different types of VEGF receptors. In some studies, the gap between VEGF and receptors is necessary for developing OHSS, and the VEGF-R2 inhibitor, like the dopamine agonist, can reverse hCG action on vascular permeability, and can also act to prevent and treat OHSS (3, 33, 34). However, other studies have associated the lower serum levels of soluble VEGF-R2 with OHSS occurrence, and the severity of the disease increases with a drop in soluble VEGF-R2. The amount of free, biologically active VEGF-A is modulated by binding to soluble VEGF-R2. Therefore, women with a large amount of soluble VEGF-R2 have a smaller quantity of free VEGF and are, therefore, at lower risk of developing severe OHSS. In contrast, women with a smaller amount of soluble VEGF-R2 exhibit higher free VEGF levels and are, therefore, at increased risk of developing OHSS (35).

## 3. Prevention 

All ovarian stimulation protocols result in some degree of hyperstimulation, but in most cases, patients do not suffer adverse consequences.

There are known factors whose presence increases the likelihood of a high response to gonadotropins; thus, the risk of developing OHSS increases: younger age, a history of a good response to gonadotropins, thin women, polycystic ovary syndrome, blood group and history of allergies (2, 36, 37). For this reason, preventive strategies begin by identifying patients at high risk of developing OHSS to individualise the ovarian stimulation protocol (gonadotropins dose, duration of FSH exposure, etc.).

Although both clinical forms of OHSS (early and late) are mediated by hCG, their onset times differ considerably. Currently, most of the measures designed to reduce OHSS are useful to minimise the early form, while the management of the late clinical form proves more difficult.

### 3.1. Early Prevention Form

#### 3.1.1. GnRH Antagonist Protocols

The use of drugs for pituitary down-regulation improves the success rates in the stimulation protocols used for IVF. These substances play an important role in lowering the incidence of a premature LH surge, resulting in lower cycle cancellation rates and improving oocyte yields with more embryos, thus allowing better embryo selection. Endogenous gonadotropin suppression was accomplished by means of gonadotropin-releasing hormone (GnRH) analogues. Two types of GnRH analogues are available: GnRH agonists (that down-regulate GnRH pituitary receptors) (38) and GnRH antagonists (that act directly and rapidly inhibit gonadotropin release) (39).

GnRH analogues act differently on the ovary. If we compare GnRH antagonists protocols with GnRH agonist protocols, we find: a) a lower exogenous FSH dose is required for ovarian stimulation due to the natural endogenous FSH’s action during the early follicular phase (40); b) less side effects related to hypoestrogenaemia (41); c) a shorter treatment cycle and reduced FSH consumption; d) lower OHSS incidence of compared to GnRH agonists (42) with a 50% reduction in the relative risk of severe OHSS (43). In addition to this, interventions such as coasting and cycle cancelation to avoid OHSS were significantly lower in the GnRH antagonist protocol (43, 44).

In spite of these advantages relating to ovarian stimulation, the probability of clinical pregnancy with GnRH antagonists initially seemed lower than with GnRH analogues (43). A very recent meta-analysis (45) was unable to provide any evidence for a difference in the live birth rates between using GnRH antagonists if compared to long GnRH agonists protocols. This indicates that similar results can be achieved with these two approaches in an adequate learning curve.

#### 3.1.2. Coasting

Coasting has been employed in ovulation induction since the 1980s to prevent OHSS in IVF, and it was the first line of intervention of choice to reduce the risk and severity of OHSS in at-risk patients (46). Coasting consists in the complete discontinuation of exogenous gonadotropin, while the administration of the GnRH analogue continues until oestradiol levels reach a plateau or lower to a safer level. The use of coasting is based on the assumption that lower FSH levels cause atresia of the small follicles, while larger follicles maintain their growth at the same time. The first published reports revealed that fertilisation and pregnancy rates were acceptable in comparison to other cycles without coasting. Yet although coasting lowered the incidence of OHSS in high-risk patients, it did not totally avoid the risk of OHSS (47).

Despite coasting being used to avoid OHSS in patients at risk, there was no sufficient evidence in the literature to determine whether coasting was an effective strategy for preventing OHSS since most published studies were retrospective and included very few cases.

Most studies conducted focused on the effect that coasting had on the GnRH agonist protocol(47). Nowadays, the widespread use of GnRH antagonists has led to the clinical practice of coasting, even with GnRH antagonists.

One of the hypotheses put forward is that the events leading to the development of OHSS are almost always related to elevated oestradiol concentrations. Coasting attempted to reduce serum oestradiol to achieve safer levels, but the time in which this is achieved is important. A significant decrease in the implantation rate was reported when coasting lasted 4 days or more. With GnRH antagonist coasting, the oestradiol concentration could be rapidly reduced to a safe level without adversely affecting oocyte maturation, fertilisation rates or embryo quality (48).

In 2007, Aboulghar (49) published the results of a randomised clinical trial which compared coasting with GnRH antagonist administration. Patients were initially stimulated by following the GnRH agonist protocol, and women at risk of OHSS (≥ 20 follicles and oestradiol concentration ≥ 3000 pg/mL) were randomised based on the following groups: Group A: the coasting protocol, consisting in continuing with the GnRH agonist and stopping gonadotropins; Group B: the GnRH agonist was discontinued by the start of the GnRH antagonist and gonadotropins doses were reduced. In the antagonist arm (Group B), the mean number of oocytes retrieved was significantly higher than in the coasting group (Group A) (16.5 ± 7.6 versus 14.06 ±5.2, P = 0.02), along with the mean number of high quality embryos (2.87 ± 1.2. versus 2.21 ± 1.1, P = 0.0001). No significant difference was found in the clinical pregnancy and multiple pregnancy rates between the groups. Surprisingly, no women developed severe OHSS in either group, but there is no data available on soft or moderate OHSS. In previous studies published by the same group, OHSS incidence was only 0.001 for the study population and 0.01 for women at risk (50).

At a later date, a retrospective case control study published by Farhi (51) compared coasting in the GnRH agonist and the GnRH antagonist protocol in hyper-response patients. None of the GnRH antagonist cycles required more than 2days of coasting, whereas 5.8% of the agonist GnRH cycles required 3days of coasting or more. Longer coasting was associated with a significant decrease in the number of oocytes retrieved in the antagonist group (2 days of coasting compared with 1 day of coasting), but the same cannot be stated for the GnRH agonist group. The authors did not find differences in the number of retrieved oocytes, fertilisation rates, high quality embryos and pregnancy rates. The moderate-severe OHSS incidence after coasting was similar in both groups (4.6% in the GnRH agonist group and 4.4% in the GnRH antagonist group).

In 2011, a Cochrane review reported no differences in the incidence of moderate or severe OHSS irrespectively of coasting being used or not. There was no difference in the clinical pregnancy rate and significantly fewer oocytes were retrieved with coasting. The problem lies in the fact that the review included only four studies, all of which were very different to each other (two studies compared coasting with unilateral follicular aspiration (52), one compared coasting with the replacement of the GnRH agonist with a GnRH antagonist (49), and the last one compared coasting versus no coasting (53). For this reason, the authors concluded that there was no evidence to suggest the benefit of using coasting to prevent OHSS if compared to not using it (or other interventions), and clinicians should use other strategies to lower the incidence of severe OHSS (54, 55).

#### 3.1.3. The GnRH Analogue: the Agonist and Antagonist in the Luteal Phase

##### 3.1.3.1. The GnRH Agonist in the Luteal Phase

One of the strategies used to prevent OHSS has been to continue GnRH agonist administration immediately after hCG administration.

The first studies were published in 1992 by Wada et al. They evaluated OHSS incidence after continuing the GnRH agonist for two weeks after the administration of 10,000 IU of hCG following the elective cryopreservation of embryos. The GnRH agonist did not prevent OHSS, probably due to the high hCG administration dose (10,000 IU). Both groups reported severe OHSS (56).

Later, another group worked on the hypothesis that GnRH agonist action may be a direct effect to reduce the VEGF expression in the ovary (57). Other studies by the same group found that the GnRH agonist directly suppressed the luteal VEGF mRNA expression during luteal formation, resulting in statistically significant reductions in VEGF, VEGF-R1, VEGF-R2, and also diminished vascular permeability in hyperstimulated rats (58).

The authors also evaluated the efficacy of continuing with the GnRH agonist for 1 week after administering 5,000 IU hCG on the risk of OHSS if compared with those patients undergoing an elective cryopreservation of all the embryos. No patient in the GnRH group developed severe OHSS (57). All the studies were no randomised, and in most studies, high gonadotropin doses were used as ovarian stimulation protocols, as evidenced by some stimulations parameters like gonadotropins doses or the oestradiol level on the day of HCG.

##### 3.1.3.2. The GnRH Antagonist in the Luteal Phase

Comparably, more recent studies have shown that the GnRH antagonist lowers the VEGF concentrations in human granulosa lutein cell cultures (59), as well as the expression of VEGF and VEGF-R in the ovaries of hyperstimulatedrats (60). However, the GnRH analogues’ mechanism of action in the ovary is not accurately known.

The first report showing the re-initiation of the GnRH antagonist on day 3 after oocyte retrieval, combined with the cryopreservation of all the embryos, was published by Lainas in 2007 (61). In this case, there were three patients at risk of OHSS and, for this reason, they were stimulated with the GnRH antagonist, but the poor ongoing pregnancy rates published with the GnRH agonist triggering led to the selection of hCG for triggering purposes. All of them improved patients’ symptoms, as well as the ultrasound and laboratory findings after the re-initiation of the GnRH antagonist. The GnRH antagonist is reported to have a prominent luteolytic effect (59), which might prove to be an alternative way of reducing the excessive production of vasoactive cytokines from the corpora lutea responsible for OHSS development.

The same group reported the combination of luteal GnRH antagonist administration and fresh blastocyst transfer in three patients with early-stage OHSS. On the day of embryo transfer (6 days after oocyte retrieval), the GnRH antagonist was re-initiated, and was administered together with oestradiol and progesterone for 4 days. Two patients accomplished pregnancies which resulted in three live healthy newborns, while a biochemical pregnancy was reported for the other patient. In all the patients, severe OHSS regressed to a moderate form of the syndrome and no pregnancy-induced severe OHSS was observed (62).

A pilot study conducted with oocyte donors and a GnRH antagonist protocol evaluated the possibility of preventing OHSS by increasing the daily GnRH antagonist dose (twice per day) before ovulation triggering (hCG) in patients at risk of OHSS (63). This study postulated that high absolute or rapidly rising serum oestradiol concentrations were associated with OHSS, while lower serum oestradiol concentrations may reduce the likelihood of OHSS (64, 65). Therefore, strategies such as the GnRH antagonist which reduces oestradiol levels could prevent early OHSS in high-risk patients. In this study, all the donors were checked on day 3 after oocyte retrieval and none of the cases developed early-stage OHSS.

The luteolytic effect of the GnRH antagonist has been proposed as the main theory to explain the mode of action of these drugs to prevent OHSS (59), although the normal luteal phase of the donors in this study does not support this idea. The authors’ hypothesis is that follicles with higher follicular oestradiol concentrations are more resistant to the influence of GnRH antagonists (66), which is based on previous studies into the polycystic ovarian syndrome where the follicles recruited at different growth phases displayed different sensitivities to GnRH antagonist administration. Hence when the GnRH antagonist dose increased, oestradiol production from the follicles was disturbed, thus establishing a dose-dependent relationship between the GnRH antagonist and high oestradiol-producing follicles.

In a randomised study, our group recently compared utilising the GnRH agonist and the antagonist immediately after HCG administration in oocyte donors who were at risk of OHSS (67). In our study, women were tested on days 3, 6 and 9 of post-oocyte retrieval. We found that the use of both GnRH analogues (especially antagonists) led to a faster recovery from ascites and to reduced ovarian size. However, no significant differences were found in terms of the incidence or intensity of the clinical manifestation, probably due to the small sample size. More studies are needed to understand the real effect of the GnRH analogues in the luteal phase to prevent OHSS. Therefore, this intervention cannot be recommended for routine use until larger studies become available.

#### 3.1.4. GnRH Agonist Triggering

HCG has been the gold standard for ovulation triggering for decades thanks to its structural and biological similarities to LH; in fact both bind to and activate the same receptor (9). The main difference between them is that hCG has a longer half-life (more than 24 hours), which may induce increased OHSS incidence (46).

The idea that the GnRH agonist trigger could eliminate OHSS in high-risk patients was introduced before the GnRH antagonist era. Some studies were published which proposed GnRH agonist triggering to be an effective alternative to hCG for inducing follicular maturation, with the potential benefit of preventing OHSS in gonadotropin-only cycles (67). Not surprisingly, the GnRH agonist trigger did not attract much interest until the GnRH antagonist era (68, 69).

When GnRH antagonist protocols were introduced for the prevention of a premature LH surge, it was possible to trigger final oocyte maturation and ovulation with a bolus of GnRH as an alternative to hCG. The GnRH antagonist binds the GnRH receptor without causing a down-regulation, and once the GnRH analogue replaces the GnRH antagonist from the receptor, it is activated which, in turn, induces the release of gonadotropins (flare up) (68).

Even though the GnRH antagonist protocol is associated with a significant reduction in OHSS incidence, OHHS cannot be excluded when ovulation has been triggering with hCG. Preliminary reports have confirmed the GnRH agonist trigger’s ability to prevent OHSS in the GnRH antagonist protocol in high-risk patients (70).

Evidence from observational uncontrolled trials and randomised studies in the last decade in OHSS high-risk patient populations suggests that the GnRH agonist for ovulation triggering significantly reduces, or even eliminates, the incidence of OHSS (71-75). In fact, no other prevention strategy even comes close to this result (76).

In the oocyte donation programme, variables such as the proportion of mature oocytes, fertilisation rates, implantation and pregnancy rates in the recipient after GnRH agonist triggering versus hCG triggering have been analysed by means of randomised clinical trials (77-79); no differences were found in these variables. OHSS was not reported after the GnRH agonist, whereas the incidence after hCG triggering was between 4% and 17% (76). Apart from the elimination of OHSS, additional benefits include a shorter luteal phase (4-6 days), reduced ovarian volume and diminished abdominal distension (72, 80).

Despite the good results for avoiding OHSS, the first reports from prospective randomised clinical trials which explored the reproductive outcome after GnRH analogue triggering revealed a poor clinical outcome when the GnRH agonist was used, in addition to a high early pregnancy loss rate despite supplementing the luteal phase with progesterone and oestradiol. The GnRH agonist has a combined negative effect on the function of both the corpus luteum and the endometrium (81, 82). The negative outcome was due to lutheal phase insufficiency in relation to low endogenous circulating LH levels (42). This was supported by good live birth rates in frozen-thawed embryo replacement cycles in which the embryos were derived from GnRH analogue-triggered cycles (71).

However following these first disappointing reports, several randomised studies now report modified luteal phase rescue with interesting results. This has been proved with intramuscular progesterone combined with oestradiol patches (80, 83), or with 1,500 UI of hCH upon oocyte retrieval or with low-dose hCG after oocyte retrieval (42, 84) with encouraging results to overcome the luteal phase and pregnancy rates without increasing the risk of OHSS (76). Further research into protocols for luteal phase supplementation after a GnRH agonist may help find the most optimal protocol.

#### 3.1.5. Other Strategies

Many strategies have been considered to prevent OHSS, including dopamine agonists (27, 85, 86) intravenous albumin administration around the time of oocyte retrieval (87, 88), glucocorticoids (89), ovarian surgery (88) and cycle cancellation.

A number of clinical trials have recently tested the clinical usefulness of a dopamine agonist as a possible way to reduce OHSS incidence and severity (3, 33, 90). A recent meta-analysis on the use of the dopamine agonist indicated that cabergoline significantly reduces the chance of developing OHSS in IVF/ICSI cycles. Nevertheless, larger trials are necessary to show a statistical difference in the severe form of OHSS. There were no statistically significant differences found for the live birth, ongoing pregnancy, clinical pregnancy and miscarriage rates in groups with or without dopamine agonists (34, 85).

Recently a case report paper has been published in which four patients diagnosed with OHSS after oocyte retrieval were treated daily with a dopamine agonist for 7 days and with a GnRH antagonist for 2 days. In this case control, all the embryos were frozen and later transferred. In all cases, the clinical manifestations of the disease rapidly diminished. More research needs to be done to understand the action of mechanism involved and to define the optimal dosing schedule (91).

Cycle cancelation is the only guaranteed method for preventing early OHSS, but it has a negative impact on patients and physicians. On the one hand, it implies an economic impact in both private clinics, where patients pay for their treatment, and public centres, where treatment is paid by the hospital. It also represents patients’ psychological distress and is related to treatment dropout.

### 3. 2. Late Prevention Forms

Late OHSS occurs after the initial 10 day-period and correlates better with the endogenous hCG produced by an implanting embryo. This serious form of the condition can thus be avoided. Despite the numerous prevention methods available, the options to manage severe OHSS once the syndrome has been established are limited.

#### 3.2.1. Vitrification of Oocytes or Embryos

Another option for patients at high OHSS risk is the cryopreservation of embryos or oocytes, and to transfer them in subsequent cycles, regardless of it involving a GnRH agonist cycle or a GnRH antagonist cycle, the GnRH agonist or hCG triggering.

Oocyte cryopreservation could be a good option for patients at increased risk of OHSS. Excellent oocyte survival rates after vitrification support the use of oocyte cryopreservation as a routine approach (92, 93). It has also been demonstrated that fertilisation, embryo development, pregnancy rate and ongoing pregnancy rate are comparable between vitrified oocytes and fresh oocytes (94, 95). Vitrification of oocytes in patients at risk of OHSS has been tested in different trials in which ovulation triggering was performed with a GnRH agonist. The results demonstrated that oocyte vitrification not only lowered the risk of OHSS in these high-risk patients, but also showed significantly higher pregnancy rates when compared with coasting in patients at risk (96).

Regarding the data on oocyte vitrification outcomes, some groups argue that oocyte cryopreservation is unnecessary given the progress made in embryo cryopreservation and the increases noted in pregnancy rates in relation to frozen embryos. After replacing the frozen-thawed embryos, live birth rates increased by utilising vitrification for embryo cryopreservation (97). However, oocyte vitrification could be an option for couples who do not desire embryo vitrification.

In a prospective cohort trial (71) with patients at risk of OHSS and concomitant GnRH-antagonist administration, final oocyte maturation was triggered with a GnRH analogue. Both the pronucleate (2 PN) oocytes were cryopreserved by vitrification, and frozen-thawed embryos were transferred in an artificial cycle. This study is the proof of concept that the GnRH-agonist triggering of final oocyte maturation combined with elective cryopreservation of the 2 PN oocytes offers OHSS-risk patients a good chance of pregnancy, while reducing the risk of moderate and severe OHSS.

Several randomised controlled trials have obtained similar pregnancy rates regardless of using elective cryopreservation of all the embryos or fresh embryo transfers.

The good ongoing pregnancy rates results obtained could be due to increased embryo survival, but could also be related to endometrial receptivity. One proposal put forward was that excessive steroid production as a side effect of ovarian stimulation might have a negative influence on endometrial (98-100) receptivity.

For frozen embryo replacement, the protocol used for endometrial preparation could take place in a natural cycle or with exogenous oestradiol and progesterone. More trials are required to choose the best protocol for frozen-thawed embryos.

#### 3.2.2. GnRH Analogues

In the case reports published by Lainas (61), the use of a GnRH antagonist in the luteal phase achieved the regression of early established severe OHSS and the birth of healthy children. Lack of data on the safety of GnRH antagonist administration for a healthy pregnancy needs to be considered when proposed as an alternative for late OHSS management. A recent review suggests that GnRH antagonist administration is not associated with either adverse effects in pregnancy or an increased risk of congenital malformation in humans (101); nevertheless, there are no data available on GnRH antagonist use in late OHSS due to pregnancy.

#### 3.2.3. Dopamine Agonist

Our group recently published a randomised trial which assessed three oral doses of a dopamine agonist (quinagolide) for the prevention of early OHSS in IVF patients (3). The incidence of a moderate/severe early OHSS rate was significantly lower when combining all the quinagolide groups if compared with a placebo (p: 0.019; OR: 0.28 80.09-0.81). Given the incidence of ultrasound evidence of ascites during the first 9 days after hCG administration, quinagolide was able to reduce the incidence of OHSS among the patients who did not achieve a clinical pregnancy from 31% with a placebo to 11% with all the quinagolide groups combined. Among the patients who accomplished a clinical pregnancy, no significant difference was found between quinagolide and placebo in relation to the presence of any ultrasound evidence of ascites.

## 4. Conclusions

Recently, there has been enough scientific evidence to establish that there are effective measures to prevent OHSS: final GnRH antagonist protocol-based oocyte maturation and GnRH agonist triggering are two strategies that may confer an even more greatly reduced risk if used combined (Table 1 and Table 2). For this reason, we must initially identify those patients at risk of OHSS (younger age, a history of or an elevated response to gonadotropins, PCOs, a very high antral follicle count) in order to personalise the stimulation protocol.

Our OHSS prevention strategies consist in: a) a personalised stimulation protocol in at-risk patients, including a GnRH antagonist cycle and GnRH agonist triggering; b) vitrification of embryos or oocytes; c) embryo replacement in natural or artificial endometrial preparation.

**Table 1.**
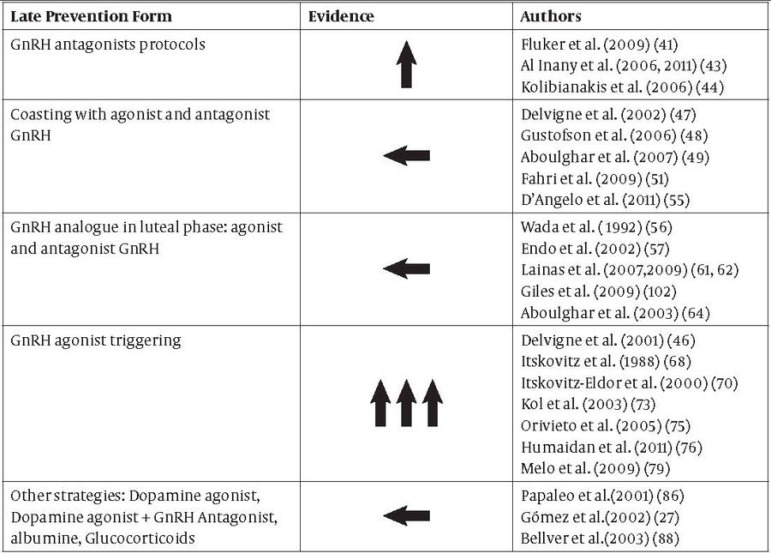
Early Prevention Form

**Table 2.**
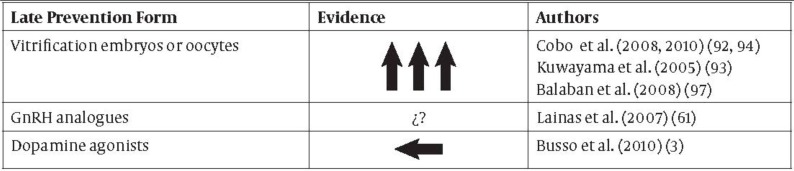
Late Prevention Form
